# Targeting mutated KRAS by HLA-A*02:01 restricted anti-KRAS TCR-mimic CAR and bispecific T cell engager

**DOI:** 10.1007/s00109-025-02585-2

**Published:** 2025-08-12

**Authors:** Saber Ebrahimi, Benedikt J. Lohnes, Shamsul A. Khan, Matthias Peipp, Ernesto Bockamp, Christian Klein, Hinrich Abken, Catherine Wölfel, Matthias Theobald, Udo F. Hartwig

**Affiliations:** 1 IIIrd. Department of Medicine – Hematology & Medical Oncology, University Medical Center, Johannes Gutenberg-University, Mainz, Germany; 2 Division of Stem Cell Transplantation and Immunotherapy, Department of Internal Medicine II, University Hospital Schleswig Holstein, Campus Christian-Albrechts-University, Kiel, Germany; 3 Institute of Translational Immunology, University Medical Center, Johannes Gutenberg-University, Mainz, Germany; 4 Roche Innovation Center Zuerich, Roche Pharma Research & Early Development, Schlieren, Switzerland; 5 Leibniz Institute for Immunotherapy, Regensburg, Germany; 6 German Consortium for Translational Cancer Research (DKTK) Partner Site Frankfurt, Mainz, Germany; 7 Research Center for Immunotherapy (FZI), University Medical Center, Johannes Gutenberg-University, Mainz, Germany; 8 Department of Biochemistry, Faculty of Chemistry and Pharmacy, Ludwig Maximilians-University of Munich, Munich, Germany

**Keywords:** Immunotherapy, T cell therapy, Chimeric antigen receptor, TCR-mimic CAR, TCR-mimic bispecific antibody, KRAS neoepitopes

## Abstract

**Abstract:**

Mutations in the KRAS proto-oncogene, particularly at codon 12, are among the most frequent genetic alterations in various cancers, and KRAS^G12V^ accounts for about 25% of all KRAS mutations observed in lung, pancreatic, and colorectal adenocarcinomas. Despite improved treatment regimes using targeted therapy and checkpoint inhibitors, cellular immunotherapy options for KRAS-mutated cancers remain elusive. We therefore developed two TCR-mimic (TCRm) anti-KRAS^G12V^/HLA-A*02:01 chimeric antigen receptors (CARs) containing different hinge regions and, alternatively, a TCRm anti-KRAS^G12V^/HLA-A*02:01 bispecific T cell engager (BiTE) to explore immunotherapy to the highly prevalent KRAS^G12V^ neoantigen. CAR-redirected or BiTE-exposed JNL-reporter cells demonstrated potent signaling capacity upon recognition of KRAS^G12V^. Moreover, human CAR T and NK cells elicited IFN-γ release and cellular cytotoxicity upon encountering target cells pulsed with KRAS^G12V^ peptide, and the anti-KRAS^G12V^ Strep-tagII hinge CAR showed superior reactivity compared to a human IgG1-Fc hinge CAR. Similarly, a novel TCRm BiTE induced strong T cell immunity to KRAS^G12V^. In contrast, we observed only very low CAR or BITE-mediated responses to naturally presented KRAS^G12V^/HLA-A*02:01 complexes. In summary, this study demonstrates that the mutation-derived KRAS^G12V^_5-14_ peptide can be effectively targeted by TCRm CAR and BiTE-redirected T cells, suggesting that TCRm anti-KRAS^G12V^ CAR or BiTE represent promising formats to advance immunotherapy to mutated KRAS neoepitopes.

**Key messages:**

- Successful development of TCRm CAR and BiTE targeting mutated KRAS/HLA-A*02:01.
- Anti-KRAS^G12V^ TCRm CAR and BiTE induce potent immunity to KRAS^G12V^ neoepitope.
- Anti-KRAS/HLA-I TCRm CARs and BiTEs are novel therapeutics for cancer immunotherapy.

**Supplementary Information:**

The online version contains supplementary material available at 10.1007/s00109-025-02585-2.

## Introduction

Monoclonal antibody (mAb)–based antibody–drug complexes and immune checkpoint inhibition (ICI) or mAb single chain variable fragments (scFv)–derived bispecific T cell engager (BiTE) and chimeric antigen receptor (CAR) T cell therapy have impressively advanced cancer immunotherapy [1–5]. Particularly, CAR T cell therapy has revolutionized the treatment of hematological malignancies, resulting in remarkable clinical improvement for patients [6, 7]. However, immunotherapy to solid tumors faces many challenges associated with tumor heterogeneity and escape variants, an immunosuppressive tumor microenvironment, and functional exhaustion of redirected effector T cells to improve treatment [8]. Moreover, the vast majority of bona fide tumor antigens derive from the tumor mutatiome [9] and thus are presented as tumor-specific peptide neoantigens (TSAs) in the context of a given HLA allele not recognized by classical CAR and BiTE therapy.

A prominent example of TSAs are peptides derived from the Kirsten rat sarcoma viral oncogene homolog (KRAS) [10]. The proto-oncogene KRAS encodes a GTPase, which activates downstream signaling pathways for cell proliferation [11, 12], and mutations of KRAS at codon 12 or 13 result in constitutive activation of RAF-MEK-ERK and PI3K pathways [12, 13]. Thus, mutated (m) KRAS represents a cancer driver oncoprotein, and KRAS codon 12 mutations are frequently found in pancreatic ductal adenocarcinomas (PDACs), colorectal adenocarcinomas (CRCs), and non-small-cell lung cancers (NSCLCs) [14–16]. Recently, new targeted therapies have entered the clinic using specific tyrosine kinase inhibitors that bind to KRAS^G12C^ and KRAS^G12D^ [17, 18]. However, acquired resistance to these inhibitors limits sustained efficacy [19–21], and a specific inhibitor to KRAS^G12V^ is still lacking.

In addition to classical T cell receptor (TCR)–mediated recognition, one promising approach to target HLA-restricted peptide neoantigens is to identify mAb-derived single-chain variable fragments (scFvs) that bind to an epitope in the context of an HLA molecule [22, 23]. These scFvs function like a native TCR [24–26], but TCR-mimic (TCRm) mAbs exhibit a higher affinity for the antigen [27]. Therefore, novel TCRm-CAR or TCRm-BiTE formats could target the invaluable source of TSA for improving cellular immunotherapy.

In this study, we therefore developed two TCRm anti-KRAS^G12V^-CD28-CD3 CARs composed of a humanized scFv recognizing the KRAS^G12V^_5-14_/HLA-A*02:01 (hereafter termed KRAS^G12V^/HLA-A2) complex [28] linked either to a human IgG1-CH2-CH3 Fc (IgFc) [29] or a Strep-tagII (STII) hinge domain [30], and, additionally, a novel TCRm anti-KRAS^G12V^ BiTE to explore cellular immunotherapy to the KRAS^G12V^ mutation.

We show that both TCRm anti-KRAS^G12V^ CAR-redirected T and NK cells demonstrated specific IFN-γ release and cytolytic activity upon recognition of KRAS^G12V^_5-14_ peptide loaded onto HLA-A2 expressing T2 cells or KRAS^G12V^ positive tumor cells. Interestingly, the anti-KRAS^G12V^ CAR encompassed with the STII spacer domain demonstrated superior activity to the IgFc hinge region containing CAR. Studies on CAR signaling using KRAS^G12V^ CAR-redirected Jurkat cells expressing the interleukin (IL)−2-dependent NFAT-Luciferase reporter system (JNL cells) [31] confirmed these results as anti-KRAS^G12V^-STII CAR T cells exerted stronger activation signaling than anti-KRAS^G12V^-IgFc CAR-redirected JNL cells. Similarly, human T cells demonstrated high CD3-mediated signaling capacity upon KRAS^G12V^–CD3 BiTE-induced recognition of KRAS^G12V^ peptide-loaded targets and elicited strong and specific anti-KRAS^G12V^ BiTE-mediated cytotoxicity.

Surprisingly, CAR- or BiTE-redirected effectors elicited very low responses when KRAS^G12V^ expressing HLA-A2 positive tumor cell lines were used as targets. In addition, T2 cells engineered to present endogenously expressed KRAS^G12V^_5-14_ were incapable to induce T or NK cell reactivity, suggesting low expression of endogenously processed and presented KRAS^G12V^/HLA-A2 complexes, which results in insufficient activation of CAR or BiTE-redirected effector cells. These results will be discussed in the light of previously published data on the presentation of endogenous, HLA-A2-restricted KRAS^G12V^ neoantigens.

## Material and methods

### Cells and culture conditions

NCI-H441, CFPAC-1, Phoenix-Ampho, and HEK 293 T were obtained from American Type Culture Collection (ATCC, Manassas, Virginia, USA) and maintained in Dulbecco’s modified Eagle’s medium (DMEM; Gibco, Karlsruhe, Germany) supplemented with 10% fetal bovine serum (FBS), 2 mM L-glutamine, and 1% penicillin–streptomycin (P/S) (Sigma-Aldrich, Steinheim, Germany) at 37 °C and 5% CO_2_. CHO, T2, the murine RMA/S cell lines, and Jurkat-NFAT-Luciferase (JNL) reporter cells (generously provided by Dr. Christian Klein, Roche Innovation Institute, Switzerland) were cultured in RPMI-1640 medium (Gibco). For the isolation of peripheral blood mononuclear cells (PBMCs), density gradient centrifugation was performed over Ficoll-Hypaque (Sigma-Aldrich). NK-92 cells (ATCC) were cultured in Minimum Essential Medium Eagle Alpha Modification (α-MEM; Gibco), supplemented with 20% FBS, 1% P/S, and 200 IU/mL IL-2 (Proleukin, Novartis, Nürnberg, Germany), 0.2 mM inositol, 0.1 mM β-mercaptoethanol, and 0.02 mM folic acid (Sigma-Aldrich).

### Expression of the anti-KRAS^G12V^ scFv D10 on HEK cells

Synthetic sequences of the anti-KRAS^G12V^ scFvs D10 and D10.7 were obtained from BioCat (Heidelberg, Germany). PCR amplified scFv D10 was cloned into a pDisplay vector (Thermo Fisher, Dreieich, Germany) to be transfected into HEK-293 T cells using FugeneHD (Promega, Madison, USA) transfection reagent according to the manufacturer’s protocols. Approximately 60 hours (h) post-transfection, cells were collected and incubated simultaneously with 5 µg KRAS peptide-loaded HLA-A2-PE tetramer per 0.5 × 10^6^ cells at 37 $^\circ{\rm C}$ for 30 min (min). The cells were then washed twice with phosphate buffer solution (PBS), and flow cytometry was used to examine specific binding of KRAS^G12V^/HLA-A2 Tetramer to the scFv D10.

### Generation of anti-KRAS^G12V^ TCRm CAR-redirected human T cells

Two different TCRm CAR constructs consisting of an immunoglobulin chain κ (Igκ) leader sequence, a single-chain variable fragment (scFv) including a GS-linker, a human IgG-Fc (IgFc) or Strep-tag II (STII) spacer domain, a CD28-derived transmembrane domain, and CD28-CD3ζ signaling domains were generated using PCR amplified fragments and the NEBuilder HiFi DNA Assembly Cloning Kit (New England Biolabs, Ipswitch, MA, USA) (primers shown in Supplementary Table S1), and cloned into the retroviral pMXs-IRES-Puro transfer vector (RTV-014, BioCAT). Phoenix-Ampho cells were transfected to produce gammaretroviral particles as described before [32] using FugeneHD (Promega, Madison, WI, USA) as a transfection reagent. After transduction, T cells were expanded by weekly polyclonal stimulation with soluble anti-human CD28 and anti-human CD3 mAbs (BioLegend, San Diego, CA, USA) and 600 IU/mL hIL-2 (Novartis), or by antigen-specific stimulation using T2 cells pulsed with 10 µg KRAS^G12V^ peptide in the presence of 1.5 µg/mL of puromycin (Sigma-Aldrich) for CAR T cell selection.

### Expression and purification of anti-KRAS^G12V^ TCRm BiTE

PCR amplified fragments and NEBuilder Hifi DNA assembly were used to generate a TCRm BiTE encoded by an Igκ leader sequence, a hemagglutinin A epitope tag (YPYDVPDYA), the KRAS^G12V^-directed scFv D10.7, and anti-human CD3 scFv fragment (clone UCHT1, generously provided by Dr. Bert Vogelstein, Harvard Medical School, USA) fused by a short GS-linker, and a 6 × His-tag (Supplementary Table S1). Following cloning into the retroviral, pMXs-IRES-Puro vector and retroviral gene transfer recombinant BiTE was expressed in stably transduced CHO cells and purified from the culture supernatant using Immobilized Metal Affinity Chromatography (IMAC) with HisPur Ni–NTA Spin Columns (Thermo Fisher Scientific). A Nanodrop 1000 spectrophotometer (Thermo Fisher Scientific) was used to determine the concentration of fusion protein.

### Detection of TCRm CAR expression by tetramer staining and flow cytometry

To detect anti-KRAS^G12V^ TCRm CARs on the surface of T-cells, HLA-A*02:01-tetramer staining (Tetramer Shop, Kongens Lyngby, Denmark) was performed according to the manufacturer’s protocols. Briefly, 5 µL of HLA-tetramer was incubated with 100 µM respective peptides for 30 min on ice in the dark. After washing cells with FACS stain buffer (PBS, 3% BSA, 1% sodium azide), HLA-tetramer was incubated with the cells for 15 min at 37 °C. Cells were washed twice with FACS stain buffer to be resuspended in 100 µL of fixation buffer (PBS, 0.4% paraformaldehyde). The intracellular staining of CHO cells was performed using the Inside Stain Kit (Miltenyi Biotec, Bergisch Gladbach, Germany) according to the manufacturer’s recommendations. Isotype controls were used throughout all staining procedures as a control for the indicated antibodies (Supplementary Table S2). Flow cytometry analysis was performed using FACS Canto II and FACS Lyric (BD Biosciences, Franklin Lake, NJ, USA), and the data were analyzed using FlowJo (Version 10.0.7) (FlowJo, Ashland, Oregon, USA).

### HLA stabilization and BiTE-binding assays using KRAS-peptide pulsed T2 cells

HLA-A*02:01 restricted KRAS^WT^ (KLVVVGAGGV) and KRAS^G12V^ (KLVVVGA**V**GV) peptides (JPT, Berlin, Germany) were diluted to a concentration of 2 µg/µL and dissolved in DMSO. For peptide-pulsing, 0.5 × 10^6^ T2 cells were incubated in serum-free RPMI-1640 with 50 µg/mL peptide and 10 µg/mL human β2 microglobulin (US Biological, MA, USA) for 18 h, washed with FACS buffer, and stained with 5 µL anti-HLA-A2-PE mAb (BioLegend) prior to resuspension in 100 µL fixation buffer. In addition, 5 × 10^5^ cancer cells or peptide-pulsed T2 cells were incubated with 10 µg/mL BiTE at 4 °C for 1 h. BiTE binding was detected using an anti-HA-Tag mAb (BioLegend) followed by flow cytometry analysis.

### Generation of endogenously KRAS peptide presenting T2 cells

The PCR-amplified KRAS^G12V^ peptide-coding sequence (Sigma-Aldrich) was cloned into the retroviral PresentER-minigene vector (Supplementary Fig. S1) and transfected into Phoenix-Ampho cells using FuGeneHD (Promega) as transfection reagent. Retroviral particles were harvested after 48 h, followed by spinoculation of T2 cells in 12-well plates at 2000 × g for 90 min at 32 °C in media containing polybrene (10 µg/mL). Accordingly, RMA/S cells were transduced with a presenter vector encoding the SIINFEKL-peptide as control. The next day, the culture medium was replaced, and cells were selected using 1 µg/mL puromycin. PresentER transduction efficiency was determined by immunofluorescent microscopy with a Texas-Red filter at a magnification of × 40 or by flow cytometry analysis of the mCherry reporter gene.

### Generation of luciferase-expressing target cells

Transduction of T2, T2presentER, NCI-H441, and CFPAC-1 cells with firefly luciferase (FLuc) was carried out as described previously [33]. Briefly, a modified pLenti-EF1a-pac-T2A-eGFP/FLuc vector encoding an FLuc/GFP fusion protein was used to generate the stable cell lines. Production of lentiviral particles was carried out by transfecting HEK-293 T cells with 3 µg of pLenti transfer vector, 1.8 µg of psPAX2, and 300 ng of pMD2.G (both from Addgene) using TransIT-LT1 transfection reagent (Mirus Bio, Madison, WI, USA). Viral supernatants were harvested after 36–48 h, resuspended in RPMI-1640 (Gibco) containing 10 µg/mL polybrene (Sigma-Aldrich), and added to target cells. A FACS Canto II (BD Biosciences) was used to determine the level of the coexpressed GFP reporter gene.

### IFN-γ ELISpot and bioluminescence-based cytotoxicity assays to examine the function of CAR and BiTE-redirected T cells

The amount of IFN-γ released upon the reactivity of anti-KRAS CAR-redirected or BiTE-mediated T cell reactivity cells was determined 24 h post coculture of effector and KRAS^G12V^ expressing target cells using the enzyme-linked immune absorbent spot (ELISpot) assay [34]. Each experiment represents technical and biological duplicates.

Cytolytic activity of anti-KRAS^G12V^ TCRm CAR T cells or TCRm BiTE-stimulated T cells against different target cells as described in the “Results” section was measured utilizing bioluminescence-based (bioluminescent, BL) cytotoxicity assays. In brief, D-Luciferin (150 µg/mL; Thermo Fisher Scientific) was added to luciferase-expressing T2 cells to measure luminescence in the absence of effector cells using a FluostarOmega-Reader (BMG LabTech, Ortenberg, Germany). A BL baseline was established to ensure equal distribution of target cells among wells. Next, either anti-KRAS TCRm CAR T cells or normal, unmodified, CD3^+^ T cells were added as effectors at various effector-to-target (E: T) ratios and incubated at 37 °C for the indicated times with BL measured as relative light units (RLU). Accordingly, CD3^+^ T cells were co-cultured with targets at different E:T ratios for indicated times in the presence/absence of the anti-KRAS^G12V^ BiTE. Target cells without effector cells were used to measure spontaneous death RLU, and 1% paraformaldehyde (PFA) (Applichem, Darmstadt, Germany) was applied to achieve maximal killing. To calculate specific cytotoxicity, the spontaneous lysis of target cells was subtracted. The percentage of lysis was determined as described before [35] as follows: 100 × (spontaneous death RLU minus test RLU)/(spontaneous death RLU minus maximal killing RLU).

### Anti-KRAS^G12V^ TCRm CAR and BiTE NFAT-FLuc reporter assays

Anti-KRAS^G12V^ TCRm CAR-expressing NFAT-FLuc JNL cells and peptide-pulsed T2 cells were cocultured in black 96-well flat-bottom tissue culture plates (Greiner Bio-One, Kremsmünster, Austria) in triplicate experiments at various effector-to-target cell ratios. Alternatively, JNL cells were cocultured with peptide-pulsed target cells, indicating the anti-KRAS^G12V^ TCRm BiTE concentration. After 24 h, the cells were harvested and centrifuged, and the supernatant was then aspirated from the cells. The cells were resuspended in 100 mL of ONE-Glo Luciferase Assay (E6110, Promega). After 30 min of incubation, the relative luminescence units (RLU) were determined using a Tecan microplate reader (Fluostar Omega-Reader).

### Statistical analysis

For statistical analysis, a two-tailed unpaired or paired Student’s *t*-tests and one-way analysis of variance (ANOVA) was used with GraphPad Prism (Version 8.4.3) with significance levels as ns (*p* > 0.12), * (*p* < 0.033), ** (*p* < 0.002), and *** (*p* < 0.001). Unless otherwise indicated, all data are presented as a mean and standard deviation (SD). Statistical analysis of differences between groups was also indicated in the figure legends.

## Results

### KRAS^G12V^ peptide pulsed T2 cells display superior expression of KRAS^G12V^/pHLA-A2 compared to naturally presented KRAS^G12V^/HLA-A2 complexes

**Fig. 1:**
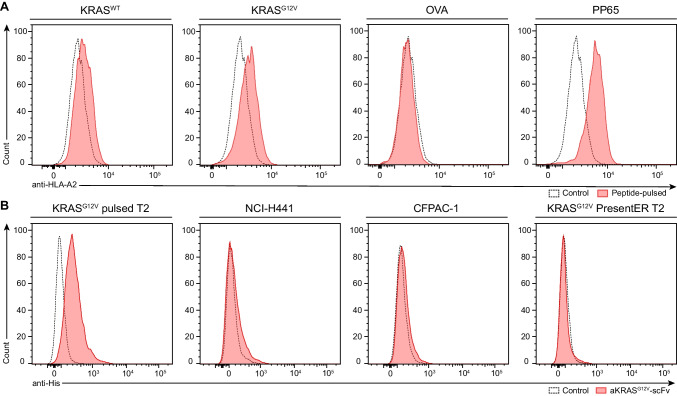
Flow cytometry analysis of peptide-HLA complex formation and scFv target binding. **A** To evaluate binding of KRAS_5-14_ peptides to the HLA-A*02:01 (HLA-A2) allel, T2 cells were peptide-loaded with KRAS^WT^ or KRAS^G12V^ (10 μg/mL) peptides. OVA^SIINFEKL^ or pp65 peptides served as negative and positive controls, respectively. HLA stabilization upon binding was analyzed compared to unloaded T2 cells (control) by quantifying the surface HLA expression using an anti-HLA-A2-PE mAb. **B** Binding of a newly generated anti-KRAS^G12V^ TCRm BiTE to KRAS^G12V^/HLA-A2 positive tumor cell lines NCI-H441 and CFPAC-1, as well as peptide-pulsed or KRAS^G12V^ presentER T2 cells, was analyzed. After incubation with BiTE, binding was detected by secondary staining with an anti-HA-tag-PE mAb. Stainings with isotype control mAb or BiTE without anti-HA-tag-PE mAb were used as controls. Flow cytometry data was acquired using a FACS Canto II

Binding of the KRAS^G12V^_5-14_ peptide to the HLA-A2 allele was previously shown. However, the stability of these KRAS^G12V^/HLA-A2 complexes was reported to exhibit short half-lives (*t*_1/2_ < 1 h) compared to control peptide [10]. We therefore investigated the relative density of peptide-stabilized KRAS^G12V^/HLA-A2 complexes in a T2 binding assay using wild-type (WT) KRAS (KLVVVGAGGV) or KRAS^G12V^ (KLVVVGAVGV) peptide-pulsed T2 cells [36]. Following staining with an anti-HLA-A2 mAb or novel generated TCRm anti-KRAS^G12V^–anti-CD3 BiTE, flow cytometry analysis revealed increased KRAS peptide-mediated HLA-A2 surface expression overall comparable to expression levels measured on HLA-A2 restricted CMV pp65_495-503_ peptide pulsed T2 cells used as controls (Fig. 1A, B), suggesting stable KRAS^G12V^/HLA-A2 cell surface complexes upon peptide binding. In contrast, no HLA-A2 expression was detectable on T2 cells pulsed with the control peptide OVA_257-264_ restricted by H-2K^b^ (Fig. 1A) confirming specificity.

In addition to peptide-pulsed T2 cells, we examined the expression levels of natural mKRAS/HLA-A2 on KRAS^G12V^ positive NCI-H441 (NSCLC) and CFPAC-1 (PDAC) tumor cells using the TCRm anti-KRAS^G12V^–anti-CD3 BiTE (Supplementary Fig. S2 and described below). The binding of BiTE to KRAS^G12V^ peptide-expressing targets detected by anti-HA-tag-PE mAb staining and flow cytometry revealed significantly reduced levels of naturally expressed KRAS^G12V^/HLA-A2 on NCI-H441 and CFPAC-1 tumor cells when compared to peptide-loaded T2 cells (Fig. 1B). Moreover, we could not detect measurable amounts of KRAS^G12V^/HLA-A2 on T2 cells engineered to present endogenously synthesized KRAS^G12V^ neoantigen by HLA-A2 using the “PresentER” antigen platform (henceforth referred to as T2presentER cells) [37] (Fig. 1B; Supplementary Fig. S3). By contrast, expression of PresentER^SIINFEKL^/H-2Kb on RMA/S control cells was clearly detectable by anti-OVA^SIINFEKL^ mAb and flow cytometry (Supplementary Fig. S3). Thus, these data suggested that either the expression level of endogenous KRAS^G12V^/HLA-A2 was very low or of limited peptide/HLA-complex stability.

### Cell surface expressed scFv D10 exhibits highly specific recognition of the KRAS^G12V^/HLA-A*02:01 complex

**Fig. 2:**
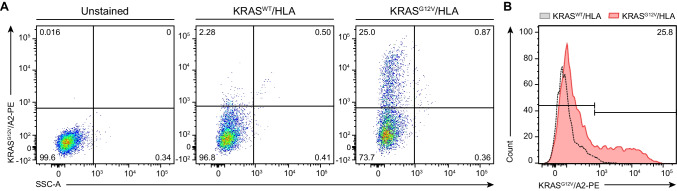
Binding specificity of anti-KRAS^G12V^/HLA-A2 scFv. **A** To investigate the binding specificity of the anti-KRAS^G12V^ scFv D10, HEK-293 T transfectants expressing D10 on the cell surface were exposed to KRAS^WT^ or KRAS ^G12V^ peptide-loaded HLA-A2-PE tetramer. Binding of the Tetramer was measured by flow cytometry analysis using unstained or MHC tetramer isotype samples as controls (not shown). **B** Flow cytometry of anti-KRAS^G12V^/HLA-A2 scFv-presenting HEK cells, stained with the KRAS^WT^ or KRAS^G12V^ peptide-loaded HLA tetramer, is depicted as an overlay histogram

In order to generate an anti-KRAS^G12V^ TCRm CAR utilizing the KRAS^G12V^/HLA-A2 recognizing scFv D10 [28], we further tested for specificity by expressing the scFv coupled to a HA-tag on the surface of HEK-293 T cells using a pDisplay vector system (Supplementary Fig. S4). Following transient transfection, staining with a mAb recognizing the HA-Tag revealed robust scFv surface expression on HEK 293 T cells as determined by flow cytometry (Supplementary Fig. S4). Next, using KRAS^WT^ or KRAS^G12V^ peptide-loaded, PE-conjugated HLA-A2 tetramer, flow cytometry analysis was performed to assess the binding specificity. While only 2.3% of unspecific binding to the KRAS^WT^ peptide was observed, 25% of KRAS^G12V^-loaded tetramer bound to the scFv could be detected (Fig. 2A, B), confirming specific recognition of the KRAS^G12V^/HLA-A2 complex.

### KRAS^G12V^ TCRm CARs expressing different spacer domains display comparable cell surface expression levels but exert different signaling capacity

**Fig. 3:**
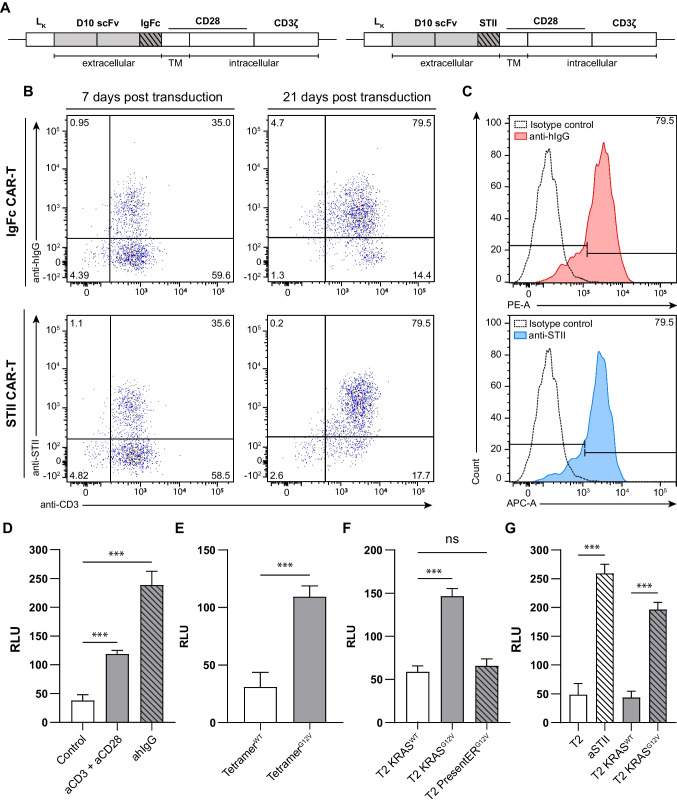
Comparable KRAS^G12V^-IgFc and KRAS^G12V^-STII CAR expression on redirected human T but differential CAR signaling capacity in transduced JNL cells. CAR constructs containing either a IgFc or STII hinge region were used to establish CAR-redirected human T and JNL cells to be tested for CAR expression and signaling ability, respectively. **A** Schematic illustration of generated CAR constructs. **B** Cell surface CAR expression was determined 7- and 21-days post-transduction by flow cytometry, using an anti-hIgG-PE mAb recognizing the CH2-CD3 Fc domain and an-anti STII-Biotin mAb staind with SA-APC. **C** Intensity of CAR surface expression was visualized as an overlay histogram compared to an isotype control sample. Flow cytometry data was acquired using a FACS Canto II. **D**–**G** ONE-Glo T cell activation assays were performed using different stimuli to investigate the signaling capacity of the CAR redirected JNL cells. **D** Polyclonal (using anti-CD3 and anti-CD28 mAbs) or anti-hIgFc mAb triggered stimulation of JNL reporter cells was used as positive controls to validate the assay. **E**–**F** Analysis of the KRAS^G12V^/HLA-A2-dependent activation was performed by incubating KRAS^G12V^-IgFc CAR JNL reporter cells with KRAS^WT^ or KRAS^G12V^ peptides loaded on **E** HLA-A*02:01 tetramers or **F** on T2 cells. PresentER^G12V^ T2 transfectants were used for endogenous peptide presentation. **G** KRAS^G12V^-STII CAR T cells were stimulated using either an anti-Strep-tag mAb or by recognition of peptide-pulsed T2 cells for ONE-Glo activation analysis. Luminescent signals were measured after 24-h incubation at 37 °C using a FLUOstar Omega plate reader (BMG LabTech). Mean values with error bars indicating the standard deviation (SD) of two technical replicates are shown and represent two independent experiments, analyzed by two-tailed unpaired Student’s *t*-test with Significance levels indicated as ns (*p* >.12), * (*p* <.033), ** (*p* < 0.002), and *** (*p* < 0.001)

Since different spacers can have a major impact on CAR functionality [38, 39], we next generated two different anti-KRAS^G12V^ TCRm CARs by fusing the anti-KRAS^G12V^/HLA-A2 scFv D10 either to a human IgG1-Fc (IgFc) [29] or a Strep-tag II (STII) [30] hinge region followed by CD28 transmembrane and costimulatory domains and the CD3ξ domain (Fig. 3A). Upon retroviral transduction, human CD3^+^ T cells were initially expanded by polyclonal (CD3/CD28) stimulation followed by repetitive antigenic specific stimulation using KRAS^G12V^_5-14_ peptide-loaded irradiated T2 cells. Subsequent flow cytometric analyses revealed expression rates of 35% and 79%, respectively, for both anti-KRAS^G12V^-IgFc and KRAS^G12V^-STII TCRm CARs in redirected T cells 7 and 21 days post retroviral gene transfer (Fig. 3B, C). Moreover, CAR expression was stable over several weeks (data not shown), suggesting that the different spacers did not influence CAR expression levels and stability.

In addition to human T cells, we further introduced the KRAS^G12V^-IgFc and KRAS^G12V^-STII CARs into the Jurkat NFAT-Luciferase (JNL) reporter cell line to examine CD3ξ signaling ability of the two CARs [31, 40]. Confirming our previous data in primary T cells, flow cytometry analysis revealed no apparent difference in JNL surface expression levels for both TCRm CARs on day 6 post retroviral gene transfer (Supplementary Fig. S5).

We then stimulated JNL cells expressing the KRAS^G12V^-IgFc CAR either polyclonally (anti-CD3/anti-CD28 mAbs) or with an anti-IgFc epitope-specific mAb, or antigen-specifically with wildtype (WT) and mutated KRAS peptides loaded onto HLA-A2 tetramer or T2 cells. While non-specific (anti-CD3/CD28) stimulation or CAR-mediated activation by an agonistic anti-hIgG mAb validated the assay and resulted in increased luciferase activity (Fig. 3D), we observed significant NFAT-induced luciferase activity with KRAS^G12V^/HLA-A2 tetramer or KRAS^G12V^ peptide-loaded T2 cells (Fig. 3E, F). Conversely, stimulation with KRAS^WT^/HLA-A2 Tetramer or KRAS^WT^ peptide-pulsed T2 did not show apparent NFAT activity (Fig. 3E, F) suggesting strong CD3ξ-mediated specific signaling upon KRAS^G12V^ peptide recognition (Fig. 3D, E, F). Of note, in line with the results described above, KRAS^G12V^ TCRm-redirected JNL cells did not elicit any bioluminescent signal upon encountering T2 KRAS^G12V^ presentER cells.

Moreover, stimulation of KRAS^G12V^-STII CAR-redirected JNL cells using either an anti-STII mAb or KRAS peptide-pulsed T2 cells were performed. Whereas reactivity to KRAS^WT^ peptide was very low and comparable with parental T2 cells (Fig. 3G), the KRAS^G12V^-STII CAR elicited a strong luciferase response following anti-STII mAb binding. Additionally, coculture with KRAS^G12V^/HLA-A2 presenting T2 cells elicited superior activity compared to the IgFc-based CAR (Fig. 3G). These results suggest that in contrast to the IgFc hinge region (239 amino acids), the STII spacer domain (60 amino acids) enhances CAR function.

### Anti-KRAS^G12V^-STII TCRm CAR-redirected T cells confer superior reactivity to mutated KRAS

**Fig. 4:**
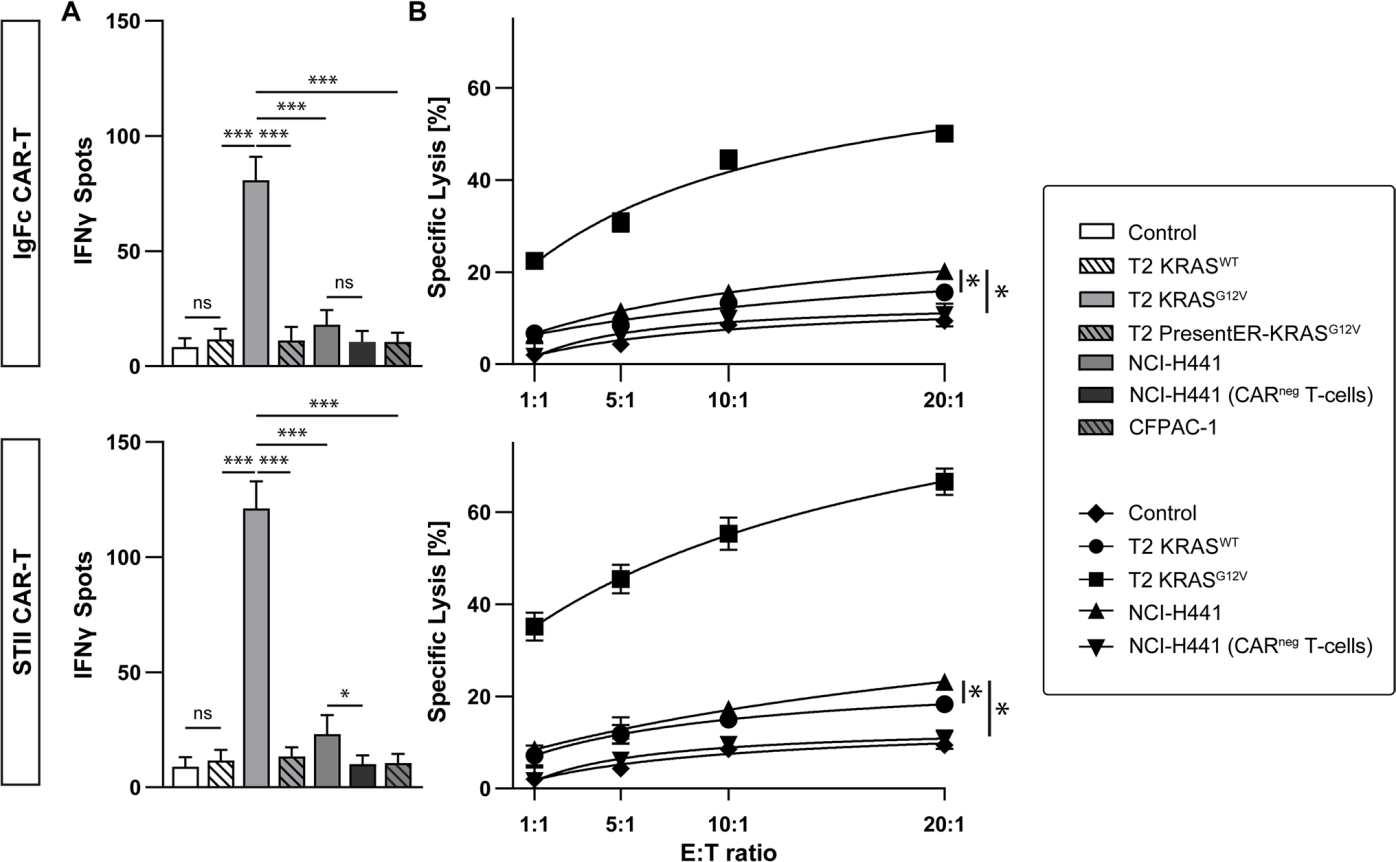
Induction of specific immunity to mKRAS by anti-KRAS^G12V^ CAR redirected human T cells. Coculture assays were performed to evaluate mKRAS-specific immune responses. **A** CAR-engineered T cells were cocultured with indicated target cell lines at an E:T-ratio of 5:1 for 24 h followed by quantifying IFN-γ release using ELISpot analysis. Non-CAR expressing T cells co-incubated with NCI-H441 tumor targets as well as CAR-redirected T cells cocultured with KRAS^WT^ pulsed T2 cells and parental T cells exposed to KRAS^G12V^ peptide-pulsed T2 cells were used as controls. Mean values with SD represent three independent ELISpot experiments. Significance of results was quantified by a two-tailed unpaired Student’s *t*-test with significance levels indicated as ns (*p* >.12), * (*p* <.033), ** (*p* < 0.002), and *** (*p* < 0.001). **B** Effector T cell cytotoxicity was assessed by coincubation of KRAS^WT^ or KRAS^G12V^ peptide-pulsed T2 FLuc^+^ cells, or the FLuc-transfected tumor cell line NCI-H441 with KRAS^G12V^-IgFc or KRAS^G12V^-STII TCRm CAR T cells at indicated E:T ratios. Accordingly, non-CAR expressing T cells co-incubated with NCI-H441 tumor targets as well as CAR-redirected T cells cocultured with KRAS^WT^ pulsed T2 cells and parental T cells exposed to KRAS.^G12V^ peptide-pulsed T2 cells were included as controls. After 12 h, cytolytic activity was determined by FLuc intensity. Mean values with error bars indicating the SD of three technical replicates are shown for one representative experiment. Significance of results was quantified by a two-tailed unpaired Student’s *t*-test with significance levels indicated as ns (*p* >.12), * (*p* <.033), ** (*p* < 0.002), and *** (*p* < 0.001)

Having demonstrated that both KRAS^G12V^ CAR formats show antigen-specific signaling capacity, we next evaluated their potential to induce inflammatory cytokines and cytotoxic activity in redirected T cells upon KRAS^G12V^_5-14_ peptide recognition. Following co-incubation of TCRm KRAS CAR-redirected T cells with either KRAS^WT^ or KRAS^G12V^ peptide loaded T2 cells at an E/T ratio of 5:1 for 24 h, each CAR engineered T cells elicited an IFN- γ response to the mutated but not WT KRAS peptide (Fig. 4A). Interestingly, KRAS^G12V^-STII CAR T cells exhibited superior IFN-γ release compared to KRAS^G12V^-IgFc CAR T cells as measured by ELISPOT assay (Fig. 4A). In contrast, no reactivity of control T cells exposed to KRAS^G12V^ peptide pulsed T2 cells and CAR T cells to T2presentER, KRAS^WT^ pulsed T2, and the KRAS^G12V^ HLA-A2 positive tumor cell line CFPAC-1 was detectable (Fig. 4A) [41, 42]. Moreover, upon co-incubation of non-CAR engineered T lymphocytes and CAR expressing T cells with KRAS^G12V^ NCI-H441, we observed only low-level IFN-γ release by both anti-KRAS^G12V^-IgFc and anti-KRAS^G12V^-STII CAR T cells (Fig. 4A) [41, 42], suggesting a putatively low concentration of KRAS^G12V^/HLA-A2 complexes expressed on the NCI-H441 tumor target.

Similarly, bioluminescent cytotoxicity assays following co-culture of CAR-redirected T cells with KRAS peptide–loaded FLuc^+^ T2 cells as well as FLuc expressing NCI-H441 and CFPAC-1 tumor cells at different E:T ratios for 12 h revealed that KRAS^G12V^-STII CAR T cells elicited up to 70% cytotoxicity to KRAS^G12V^ peptide–loaded T2 targets at an E:T ratio of 20:1, while KRAS^G12V^-IgFc CAR T cells reached 60% cytolytic activity at the same E:T ratio (Fig. 4B). In support of the IFN-γ release data, KRAS^G12V^-STII CAR T effectors demonstrated a low (25%) but significant cytolytic reactivity to NCI-H441 at an E:T ratio of 20:1 when compared to CAR-negative (CAR^neg^) T cells and CAR-redirected T cells exposed to KRAS^WT^ peptide pulsed T2 cells. Low cytolytic activity (20%) was also observed with KRAS^G12V^-IgFc CAR-redirected T cells as compared to controls (Fig. 4B).

Taken together, these data demonstrated that TCRm KRAS^G12V^ CAR-redirected T cells effectively induce specific release of inflammatory cytokines shown exemplarily for IFN-γ and exhibit low but significant cytotoxic reactivity to KRAS^G12V^/HLA-A2 presenting targets.

### TCRm anti-KRAS^G12V^ bispecific T cell engager elicit potent anti KRAS^G12V^-mediated T cell immunity

**Fig. 5:**
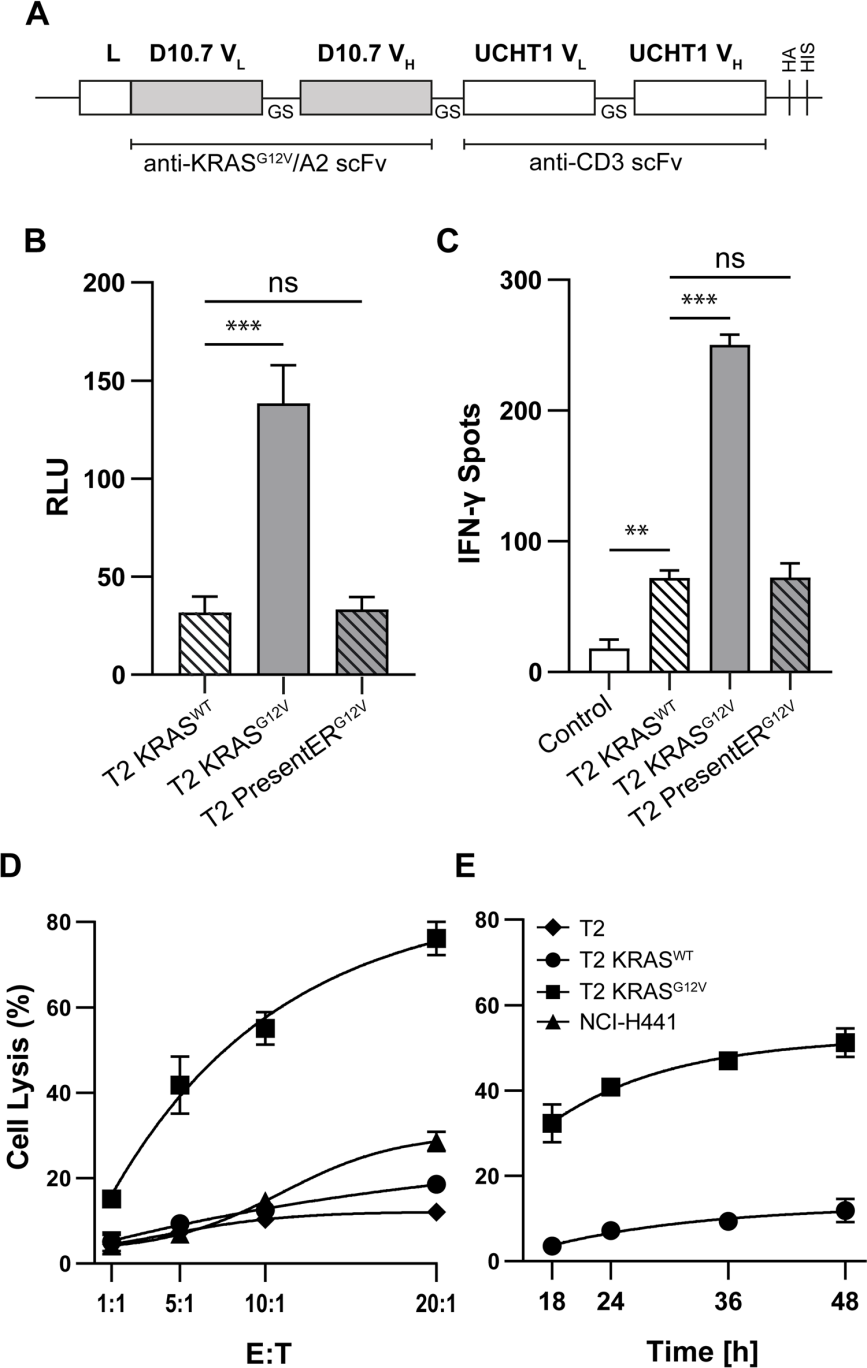
Induction of specific immunity to mKRAS by anti-KRAS^G12V^ BiTE redirected human T cells. Coculture assays of human T cells and KRAS-peptide loaded FLuc^+^ T2, or FLuc^+^ NCI-H441 target cells, were performed in the presence of anti-KRAS^G12V^ TCRm BiTE to evaluate mKRAS-specific immune responses. **A** Schematic illustration of the generated BiTE construct. **B** CD3^+^ JNL and KRAS peptide-loaded target cells were incubated at a ratio of 1:1 in the presence of 4.4 nM BiTE concentration. After 24 h of cocultivation with JNL cells, FLuc^+^ activity was analyzed using a FLUOstar Omega plate reader. One representative out of three independent experiments is shown. **C** Human T cells were cocultured with KRAS^WT^ or KRAS^G12V^ peptide-pulsed FLuc^+^ T2 and FLuc^+^ T2presentER cells, respectively, at an E:T ratio of 5:1 in the presence of 1.7 nM BiTE, and IFNγ release was quantified after 24 h by IFN-γ ELISpot analysis. T cells cocultured with non-peptide pulsed T2 cells were used as controls. Significance of results was quantified by a two-tailed unpaired Student’s *t*-test with significance levels indicated as ns (*p* >.12), * (*p* <.033), ** (*p* < 0.002), and *** (*p* < 0.001). **D** BiTE mediated cytotoxicity was determined by coincubating KRAS^WT^ or KRAS^G12V^ peptide-pulsed FLuc^+^ T2 cells or FLuc^+^ NCI-H441 with varying E:T ratios in the presence or absence of 1.7 nM BiTE for 24 h followed by measuring biolumescence intensity upon addition of luciferin. One representative out of three independent experiments is depicted. **E** Coculture of T cells and KRAS peptide loaded FLuc^+^ T2 targets at an E:T ratio of 5:1 was performed with BiTE (1.7 nM) at different time points to determine the time-dependent kinetic of KRAS^G12V^ specific cytolytic activity after addition of luciferin. Bioluminescence was detected and quantified using a FLUOstar Omega plate reader. One representative out of three independent experiments is shown

In addition to anti-KRAS^G12V^ CAR T cells, we further explored a novel TCRm BiTE recognizing mutated KRAS/HLA-A2. To this end, in contrast to the scFv D10 [28] used for generating the TCR-mimic anti-KRAS CARs, the coding regions of the anti-KRAS^G12V^ scFv D10.7 reported to have a higher affinity compared to D10 [28] and the anti-CD3 scFv derived from the clone UHCT1 [43] were assembled via a GS linker followed by sequences encoding an HA-tag and a 6xHis tag (Fig. 5A). Following lentiviral gene transfer and stable expression in CHO cells, recombinant BiTE was purified by His-Tag-based immobilized metal affinity chromatography to be functionally tested. To first examine binding to CD3 and subsequent T cell activation, anti-KRAS^G12V^ BiTE was added at 1 μg/mL to JNL cells cocultured with KRAS-peptide-loaded T2 and T2presentER targets (E:T ratio of 1:1). Strong induction of NFAT-driven luciferase activity revealed significant BiTE-mediated CD3 signaling upon recognition of KRAS^G12V^ but not KRAS^WT^ indicating profound and specific T cell activation via the TCRm KRAS BiTE (Fig. 5B). In support of earlier results, we did not detect recognition of KRAS^G12V^ T2presentER cells by T cells in the presence of BiTE (Fig. 5B). Similarly, human T cells elicited significant and specific IFN-γ response upon BiTE-mediated recognition of KRAS^G12V^ on peptide loaded T2 cells (E:T ratio of 5:1), whereas no cytokine release was found after co-incubation of T effectors with T2 targets expressing KRAS^WT^ peptide or T2presentER cells (Fig. 5B).

We next investigated the potential of the KRAS^G12V^ TCRm BiTE to induce specific cytotoxicity.

As depicted in Fig. 5C, human T cells cocultured with KRAS^G12V^-peptide presenting FLuc^+^ T2 targets for 24 h at different E:T ratios demonstrated strong BiTE-mediated specific cytolytic activity to KRAS^G12V^/HLA-A2 but not to KRAS^WT^/HLA-A2 on T2 cells at a BiTE concentration of 8.89 nM (Fig. 5C). T cells cocultured with non-peptide pulsed T2 cells in the presence of BiTE served as control (Fig. 5C). Moreover, we could measure a low (23%) but detectable antitumoral effect of BITE-induced T cell reactivity at an E:T ratio of 20:1 (Fig. 5C). In addition, extending the coculture time to 48 h (E:T ratio of 5:1) revealed that optimal TCRm BITE-mediated T cell-cytotoxicity occurred 36 to 48 h post binding of BiTE to CD3 on effector T cells (Fig. 5D).

Taken together, these results demonstrated that in addition to TCRm CARs, TCRm bispecific T cell engager exerts potent and specific cytolytic activity against the KRAS^G12V^/HLA-A2 target complex.

### KRAS^G12V^ TCRm CAR-redirected NK cells show reactivity to mutated KRAS

Since KRAS^G12V^ CAR T cells demonstrated significant reactivity to mutated KRAS, we further introduced the KRAS^G12V^ -STII CAR into the human NK cell line NK92 [44] as a proof of concept.

**Fig. 6:**
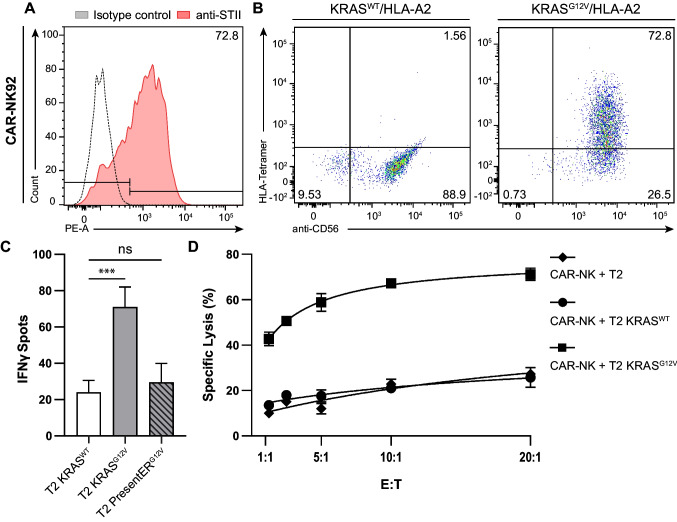
Generation and functional testing of KRAS^G12V^–STII TCRm CAR redirected NK92 effector cells. NK92 cells were transduced with the KRAS^G12V^-STII TCRm CAR construct. **A** CAR surface expression was assessed by identifying NK cells using an anti-CD56-APC mAb in combination with an anti-Strep-tagII-Biotin mAb followed by SA-PE staining. As a control, unstained samples and isotype mAb were used. **B** CAR expression was determined by staining the anti-KRAS^G12V^ TCRm CAR with PE-conjugated HLA-A2 tetramers loaded with either KRAS^WT^ or KRAS^G12V^ peptide and flow cytometry analysis. **C** To measure CAR-mediated IFN-γ release, coculture of KRAS^WT^ or KRAS^G12V^ peptide-pulsed FLuc^+^ T2 cells and KRAS^G12V^ FLuc^+^ T2 presentER cells was performed at an E:T ratio of 5:1 for 24 h followed by IFN- γ ELISpot analysis. Significance of results was quantified by a two-tailed unpaired Student’s *t*-test with significance levels indicated as ns (*p* >.12), * (*p* <.033), ** (*p* < 0.002), and *** (*p* < 0.001). **D** Following concubation of KRAS^G12V^ CAR redirected NK92 effectors with KRAS-peptide loaded FLuc^+^ T2 targets at indicated E:T ratios for 24 h, cytolytic activity was determined by bioluminescence. One representative out of three independent experiments is depicted

After retroviral transduction and expansion of NK-92 cells, flow cytometric analysis for CAR expression by either using an anti-STII-Biotin mAb followed by streptavidin-PE or KRAS^G12V^ peptide loaded HLA-A2-PE tetramer revealed that 72.8% of the transduced CD56^+^ NK92 cells expressed the CAR (Fig. 6A, B). To test for specific recognition of KRAS^G12V^/HLA-A2, we determined IFN-γ cytokine release by KRAS^G12V^ -STII CAR engineered NK92 cells upon co-cultivation with KRAS peptide-pulsed T2 by ELISpot assay. These analyses revealed a threefold increase of IFN-γ upon recognition of KRAS^G12V^ as compared to KRAS^WT^ presented by T2 cells (Fig. 6C). Of note, PresentER-KRAS^G12V^ T2 cells marginally induced secretion of IFN-γ by redirected NK92 cells upon exposure to antigen, but this response was not significant when compared to the KRAS^WT^ peptide (Fig. 6C). In addition, coculturing KRAS^G12V^-STII CAR-redirected NK92 effectors with KRAS^G12V^-peptide loaded T2 cells revealed potent and mutated KRAS-specific NK cytotoxicity of 70% at an E:T ratio of 20:1 (Fig. 6D). As the cytolytic activity observed to KRAS^WT^ peptide-loaded and parental T2 target cells by NK92 cells was comparable (Fig. 6D), this likely reflects non-specific killing due to the absence of appropriate HLA class I expression on T2 cells except for the peptide stabilized HLA-A2 allele, which results in natural NK cell cytotoxicity to targets expressing insufficient amounts of HLA class I molecules.

Taken together, these initial findings suggest that in addition to TCRm BiTE or TCRm CAR-redirected T cells, KRAS^G12V^ TCRm CAR-expressing NK cells can further induce robust immunity to mutated KRAS.

## Discussion

Particularly in tumor entities with unfavorable prognoses, such as non-small cell lung cancer and pancreatic ductal adenomas, effective and sustainable immunotherapy, ideally combined with targeted therapy or immune checkpoint blockade, is currently lacking. Given that the majority of tumor-specific neoepitopes derive from the tumor mutatiome, HLA-restricted peptide neoantigens represent attractive target structures that can be exploited for CAR T therapy. TCRm CARs composed of a peptide/HLA complex recognizing scFv [26, 27, 45, 46]. Moreover, potent antitumor immunity by TCRm CAR-redirected T cells, e.g., recognizing peptides derived from regulatory proteins overexpressed in tumors or cancer-testis antigens, has recently been reported [47–49].

Hence, as peptides derived from the KRAS^G12V^ mutation represent truly specific neoantigens in NSCLC and PDAC, and the development of a TCRm CAR recognizing mutated KRAS/HLA-A11 was recently described [50], we explored two different TCRm CARs, composed of the KRAS^G12V^/HLA-A2 complex recognizing scFv D10 [28], either linked to a human IgFc [29] or to the recently described STII spacer [30] to investigate immunity to a KRAS^G12V^_5-14_/HLA-A2 peptide complex. In addition to CARs, we further generated a TCRm anti-KRAS^G12V^–anti-CD3 BiTE using the high affinity KRAS^G12V^/HLA-A2 scFv 10.7 [28]. Confirming previously reported data [10, 42], we could first show that the originally NetMHC 4.0 predicted KRAS^G12V^_5-14_ decamer KLVVVGA**V**GV not only binds to the HLA-A2 allele upon loading onto T2 cells but is also specifically recognized by the scFv D10, as demonstrated by the expression of the scFv on HEK 293 T transfectants and KRAS^G12V^/HLA-A2 tetramer staining.

We could then show that both KRAS^G12V^ CAR constructs were expressed at comparable levels in engineered human T cells 21 days post retroviral gene transfer, suggesting that the different hinge regions did not affect CAR expression. Additionally, both KRAS^G12V^ CARs demonstrated antigen-specific signaling via the CD3ζ domain following recognition of KRAS^G12V^/HLA-A2 tetramers as examined by CAR-expressing JNL-reporter cells shown previously as a suitable system for testing the signaling capacity of TCRm CARs [31]. However, we found that constructs containing the STII hinge region were superior to those with the IgFc region. Subsequent evaluation of both CARs for their ability to exert potent effector function, examined by IFN-γ release and cytolytic reactivity upon engaging KRAS^G12V^ peptide presenting T2 target cells, confirmed that human T lymphocytes engineered to express the anti-KRAS^G12V^-STII CAR elicited superior cytotoxicity as compared to anti-KRAS^G12V^-IgFc CAR-redirected T effector cells. Using the human cell line NK92 [44] expressing anti-KRAS^G12V^-STII CAR, we could confirm that this CAR additionally induced potent NK-reactivity to mutated KRAS. Since NK cells do not elicit alloreactivity and thus in contrast to T cells do not need to be HLA-matched, redirecting NK cells with the TCRm KRAS^G12V^ CAR might represent an interesting’off-the-shelf’approach for cellular therapy.

Our results on the role of different hinge domains for TCRm CAR function are supported by previously published data showing that non-signaling spacers with short lengths can facilitate CAR-mediated T cell effector functions after target engagement [38, 39]. Furthermore, CARs containing a STII spacer sequence have been shown to induce stronger cytotoxicity compared to IgG4 hinge–based CAR-T cells [30]. However, the STII spacer consists of an eight amino acid comprising synthetic sequence (Trp-Ser-His-Pro-Gln-Phe-Glu-Lys), which might induce immunogenicity. To our knowledge, this spacer has so far not been implemented in CARs used clinically to examine this point, but screening of STII and candidate flanking sequences of the vector-backbone using the NetMHC3.4 algorithm has not resulted in identifying HLA-binding peptides likely to bind to common HLA class I alleles with high affinity [30]. In addition, strep-tag sequences are commonly used as a tag sequences in recombinant proteins such as, e.g., bispecific antibodies, and, in contrast to the 6xHis tag, are reported to be not or only weakly immunogenic.

In addition to CAR-engineered T and NK cells, we further explored the immunotherapeutic potential of a TCRm anti-KRAS^G12V^ BiTE. Considering therapy, recombinant bispecific antibody formats have the advantage to be applicable as “off-the-shelf” therapeutics. Signaling studies using JNL reporter cells, co-incubated with KRAS^G12V^ peptide loaded T2 cells, demonstrated potent T cell activation by BiTE-mediated recognition of KRAS^G12V^ peptide–loaded targets but not to KRAS^G12V^ T2presentER cells and KRAS^WT^ peptide. Likewise, BiTE induced strong and specific IFN-γ release and cytolytic reactivity in human T cells upon KRAS^G12V^ recognition, suggesting that the anti-KRAS^G12V^-STII CAR and anti-KRAS^G12V^–anti-CD3 BiTE were comparably effective. Moreover, our kinetic studies further revealed that BiTE-mediated anti-KRAS^G12V^ reactivity peaked 24 to 36 h after coculture of effector and target cells.

In addition to strong BiTE-mediated reactivity of T cells to KRAS^G12V^ peptide–loaded T2 targets, we also observed a very low response to NCI-HH441 cells, while no reactivity to the CPFAC target cell line was detectable. NCI-H441 tumor cells have been previously reported to express only nine copies of KRAS^G12V^ on their cell surface, which could still be detected by an anti-KRAS^G12V^/HLA3 specific BiTE [42], and we also observed a very low but significant cytolytic reactivity by the anti-KRAS^G12V^ TCRm CAR-redirected T cells. Hence, NCI-H441 might also express very low copy numbers of KRAS^G12V^/HLA-A2 complexes, however not sufficient to mount a substantial immune response as observed with KRAS^G12V^ peptide pulsed T2 cells. Alternatively, the response observed with the anti-KRAS^G12V^ TCRm BiTE could be conceivably due to the use of the scFv 10.7, shown to have a higher affinity compared to the scFv D10 [28] and might reflect cross-reactivity to similar peptides reported for other high-affinity scFv to mutated KRAS^G12D^ [50].

A low-level response to KRAS^WT^ peptide-presenting targets was apparent both in studies using KRAS^G12V^ CAR-engineered T and NK cells. Basal to low-level nonspecific reactivity, especially at higher E:T ratios, has been observed previously [9, 49] and can be largely attributed to the affinity and antigen selectivity of the scFv used, i.e., that higher affinity can be associated with an increased risk of cross-reactivity. Additionally, nonspecific reactivity could, at least partially, reflect alloreactive responses to peptide-stabilized HLA-A2 complexes expressed on T2 cells as the T effector and target cells were not matched for HLA-A2. Since CAR NK92 cells were co-incubated with peptide-loaded T2 cells, which do not express any other HLA class I alleles except peptide stabilized HLA-A2, reactivity examined for T2 expressing KRAS^WT^ peptide is presumably caused by “non-self” NK-reactivity driven in the absence of HLA class I as shown by the response to parental T2 cells used as controls.

Despite some low responses observed by KRAS^G12V^ CAR-redirected T cells or by anti-KRAS^G12V^ BiTE to NCI-H441, the lack of sufficient reactivity to naturally presented KRAS^G12V^/HLA-A2 on NCI-H441 and CFPAC-1 cells compared with KRAS^G12V^ peptide pulsed T2 targets clearly represents a limitation of our study. As engineered T2presentER cells expressing endogenous KRAS^G12V^ peptide to be presented by HLA-A2 did not reveal any KRAS^G12V^-specific T cell reactivity, one reason for this observation might be an insufficient stability of KRAS^G12V^/HLA-A2 complexes as suggested earlier [10]. As a consequence, we were not able to evaluate the therapeutic efficacy of the anti-KRAS^G12V^-STII TCRm CAR and anti-KRAS^G12V^ TCRm BiTE in preclinical models.

Our data are in line with controversial results published previously on the recognition of KRAS^G12V^/pHLA-A2 by T cells [51–54]. While Rive et al. described the isolation of a KRAS^G12V^_5-14_ specific TCR from PBMC-derived CD8^+^ T cells of a PDAC patient and reported KRAS^G12V^ specific antitumoral immunity following adoptive transfer of human T cells engineered to express this TCR into an intraductal papillary mucinous neoplasia (IPMN) pancreatic cancer cell engrafted CB-17 SCID PDX model [51], the group of Shen et al. reported successful antitumor activity of an anti-KRAS^G12V^ TCRm antibody–drug conjugate intravenously administrated into immunocompromised BALB/c nude mice engrafted with K562 transfectants expressing endogenous KRAS^G12V^_5-14_ presented by HLA-A2 [52]. However, the KRAS^G12V^-specific TCRs reported were not tested with parental K562 to exclude cross-reactivity to HLA alleles potentially expressed on K562 cells [51]. Furthermore, successful reactivity of the TCRm-drug conjugate was only achieved upon stable expression of a ubiquitin-KRAS^G12V^ recombination protein resulting in increased KRAS^G12V^/HLA-A2 complexes on the transfected target cell line [52].

Moreover, these data are contradicted by studies of Willimsky et al., who explored anti-KRAS^G12V^ antitumor immunity of human T cells engineered to express KRAS^G12V^-specific TCRs obtained from transgenic (ABabD9II) mice, harboring a repertoire of human HLA-A2 restricted, αβ-TCRs expressing T cells, following immunization with non-spliced and spliced KRAS^G12V^ peptides (KLVVGA**V**GV and KL/VVVA**V**GV) [53]. In support of our observations, anti-KRAS^G12V^ TCR-redirected T cells conferred only reactivity to different human KRAS^G12V^/HLA-A2 tumor cell lines when additionally loaded with KRAS^G12V^ peptide, whereas no response to endogenous KRAS^G12V^/HLA-A2 complexes could be detected irrespectively of elevated HLA-A2 expression following IFN-γ pretreatment or engineered cancer cells overexpressing KRAS^G12V^ peptide [53].

Moreover, detailed in vitro proteasome-catalyzed peptide splicing (PCPS) analyses by Mishto et al. revealed that both the KRAS^G12V^_5-14_ (KLVVVGA**V**GV) non-spliced epitope used in our study, as well as the KRAS^G12V^_5-6/8–14_ (KL/VVGA**V**GV) spliced epitope can be generated in vitro by proteasomes and are comparably transported into the ER-lumen. However, the binding affinity of KRAS^G12V^_5-14_ to HLA-A2 was found to be six-fold reduced compared to the KRAS^G12V^_5-6/8–14_ spliceotope [54]. This result was corroborated by the finding that KRAS^G12V^/HLA-A2 complexes exhibit a short half-life (*t*1/2 < 1 h) when compared to control peptides [10]. Additionally, despite recognition of both epitopes by KRAS^G21V^-specific T cell clones, none of the two peptides could be identified in the HLA-A2 immunopeptidome derived from the KRAS^G12V^ positive colon carcinoma cell line SW480 [54], further supporting our findings and the data reported by Willimsky et al. [53].

Taken together, although our results complement previous data published on the lacking immunogenicity of naturally presented KRAS^G12V^ neoepitope in the context of HLA-A2, it remains to be further elucidated whether endogenously processed KRAS^G12V^ peptides are stably expressed at sufficient density to mount an immune response in the context of HLA-A2.

In summary, our studies demonstrate that the development of both TCRm anti-KRAS^G12V^ CARs and BiTEs to KRAS^G12V^/HLA-A2 is feasible and can result in potent reactivity. However, TCRm CARs and BiTEs recognizing KRAS^G12V^ neoepitopes presented, e.g., by HLA-A*03:01 or HLA-A*11:01 will likely be better candidates for improving immunotherapy to mutated KRAS [42, 50]. Since TCRm cell-surface epitope density of a given peptide/HLA is lower than the epitope density recognized by classical, non-HLA-dependent mAbs, limitations in the efficacy of TCR-mimic therapies to KRAS^G12V^ might be improved by a new receptor format combining TCR and CAR features [55].

## Supplementary Information

Below is the link to the electronic supplementary material.

ESM 1 (DOCX 4.08 MB)

## Data Availability

The data are available from the corresponding author upon reasonable request.
