# Supplementary material for: Targeting mutated KRAS by HLA-A*02:01 restricted anti-KRAS TCR-mimic CAR and bispecific T cell engager
Source: J Mol Med (Berl). 2025 Aug 12;103(10):1231–46. doi: 10.1007/s00109-025-02585-2 (PMC12449438; doi:10.1007/s00109-025-02585-2)
Supplement: Supplementary file 1 — (DOCX 4.08 MB) [file 109_2025_2585_MOESM1_ESM.docx]

**Supplementary Information**

**Targeting mutated KRAS by HLA-A*02:01 restricted anti-KRAS TCR-mimic chimeric antigen receptor and anti-KRAS TCR-mimic bispecific T cell engager**

Saber Ebrahimi^1^, Benedikt J. Lohnes^1^, Shamsul A. Khan^1^, Matthias Peipp^2^, Ernesto Bockamp^3,6^, Christian Klein^4^, Hinrich Abken^5^, Catherine Wölfel^1,6^, Matthias Theobald^1,6,7^, Udo F. Hartwig^1,6,7^

^1^IIIrd. Department of Medicine – Hematology & Medical Oncology, University Medical Center of the Johannes Gutenberg-University, Mainz, Germany

^2^Division of Stem Cell Transplantation and Immunotherapy, Department of Internal Medicine III, University Hospital Schleswig Holstein, Campus Christian-Albrechts-University Kiel, Germany

^3^Institute of Translational Immunology, University Medical Center of the Johannes Gutenberg-University, Mainz, Germany.

^4^Roche Innovation Center Zuerich, Roche Pharma Research & Early Development, Schlieren, Switzerland

^5^Leibniz Institute for Immunotherapy, Regensburg, Germany

^6^German Consortium for Translational Cancer Research (DKTK), Partner Site Frankfurt / Mainz, Germany

^7^Research Center for Immunotherapy (FZI), University Medical Center of the Johannes Gutenberg-University, Mainz, Germany

**Correspondence to:** Dr. Udo Hartwig, uhartwig@uni-mainz.de, +496131179790


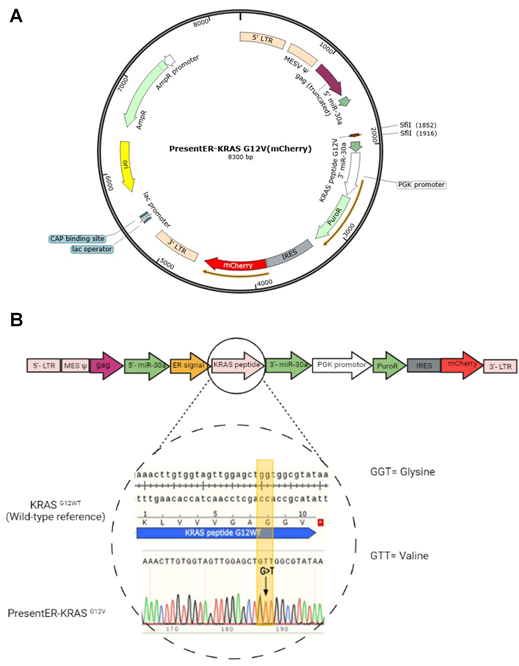


**Supplementary Figure S1.** (**A**) Schematic representation of the retroviral transfer vector PresentER encoding KRAS^G12V^ minigene sequence with an endoplasmic reticulum (ER) signal sequence. This vector contains puromycin- and ampicillin-resistance genes, an internal ribosome entry site (IRES), red fluorescent protein (mCherry), long terminal repeats (LTRs), and viral package signal (ψ). SfiI restriction sites displayed were used for the PCR cloning experiment. (**B**) Representative sequencing result of DNA vector encodes a 10mer-peptide KRAS^G12V^ compared to reference sequence encoding KRAS^G12WT^ as a wild-type peptide.

**B**

anti-KRAS^G12V^ - anti-CD3 BiTE


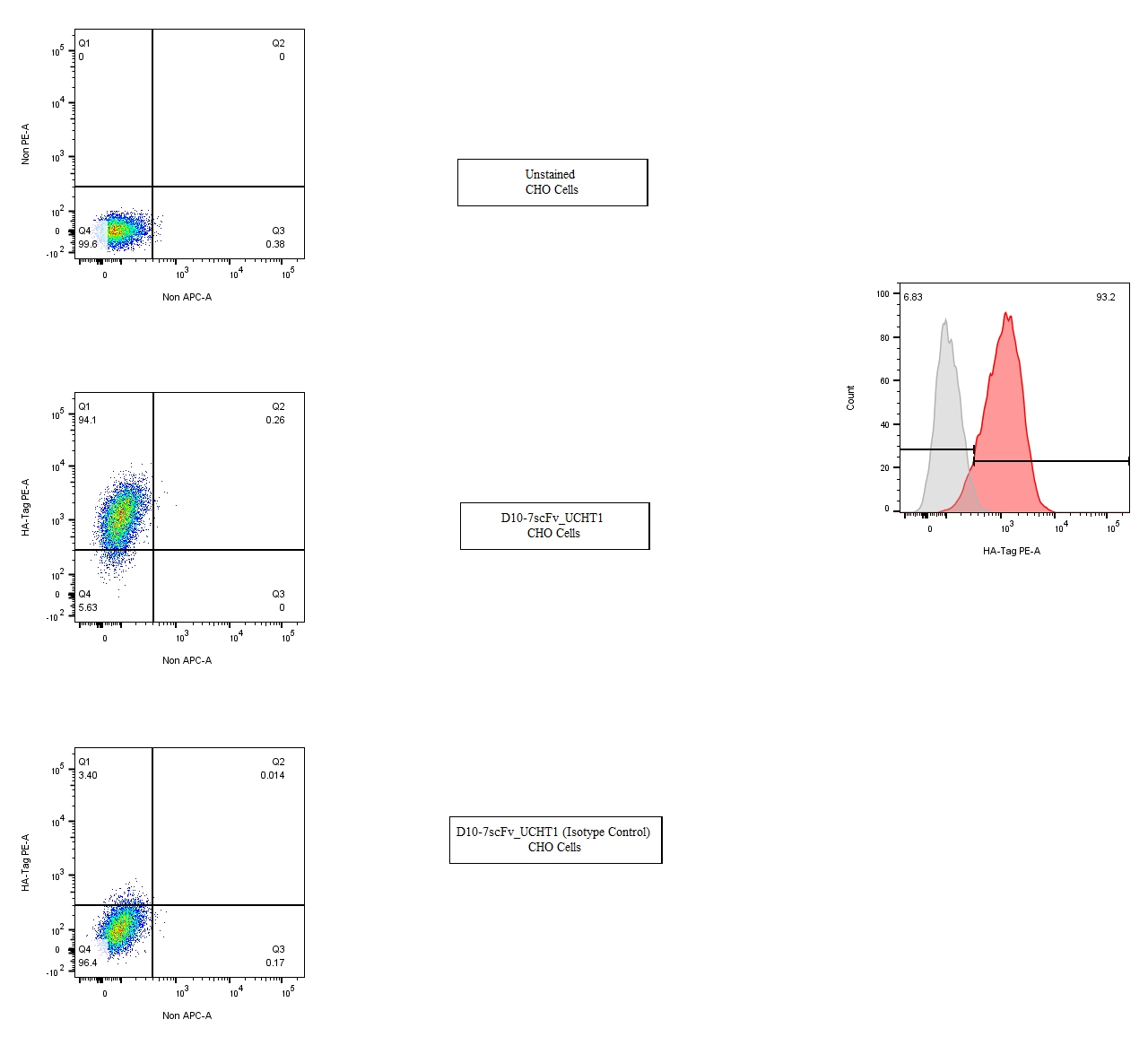


Count

PE-A

anti-HA-tag

Isotype control

**anti-KRAS^G12V^ - anti-CD3 BiTE**


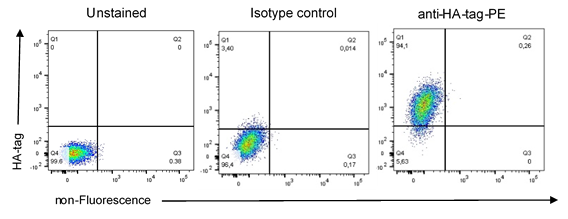


**A**

**­­**

**Supplementary Figure S2.** Flow cytometric analysis of scFv-UCHT1 expression in CHO cells. (**A**) Intracellular staining of scFv-UCHT1-expressing CHO cells with an anti-HA-tag antibody. Unstained cells and cells were only incubated with the antibody of the same isotype served as controls. (**B**) Flow cytometry data shown in (A) are presented as a histogram. The red histogram indicates transduced CHO cells stained with anti-HA-tag antibodies, whereas the gray histogram represents transduced cells stained with an irrelevant antibody as an isotype control. CHO, chinese hamster ovary cells; HA-tag, hemagglutinin-tag.

**A**

**B**


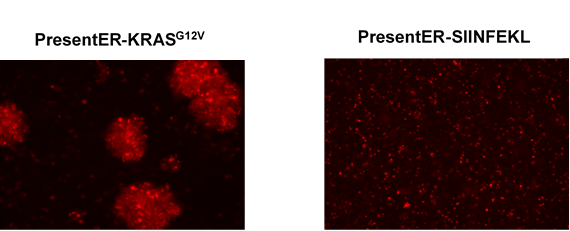

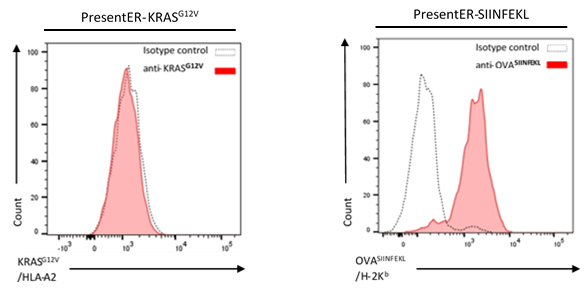


**Supplementary Figure S3. (A)** Analysis of mCherry expression in cells transduced with PresentER-minigenes. T2 cells expressing PresentER-KRAS^G12V^ (mCherry) and RMA/S cells expressing PresentER-SIINFEKL (mCherry) were visualized with a Texas-Red filter under a fluorescence microscope. (**B**) Flow cytometry analysis of cells expressing the PresentER-based minigenes.

**A**


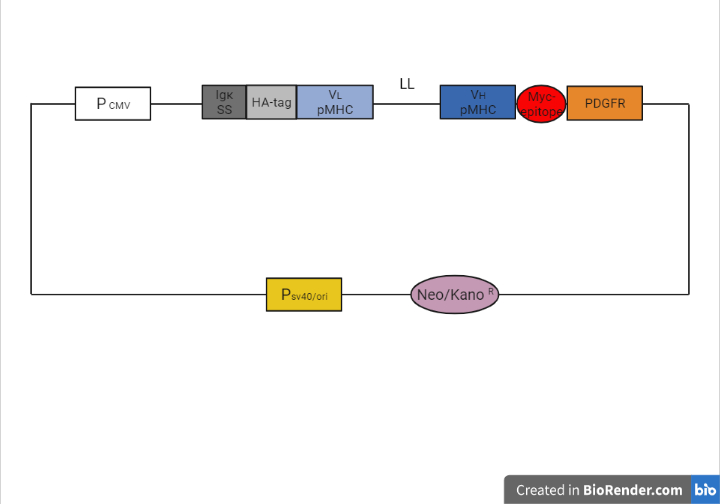


**pDisplay-(anti-KRAS^G12V^) scFv**

Count

PE-A


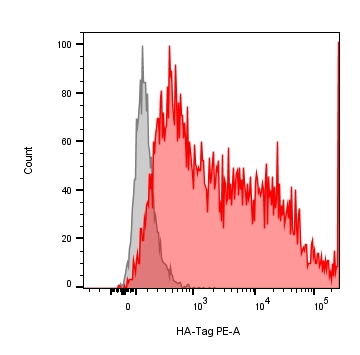


**HEK-239T**

**pDisplay-scFv**


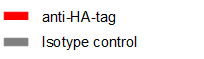


**B**

**Supplementary Figure S4. (A)** Illustration of (anti-KRAS^G12V^) scFv grafted into an expression vector pDisplay. HA-tag, hemagglutinin-tag; Igκ SS, Igκ-chain signal sequence; LL, long linker; Myc, an epitope tag; P_CMV_, cytomegalovirus promoter; PDGFR, human platelet-derived growth factor receptor; pMHC, peptide-major histocompatibility complex; P_SV40/ori_, SV40 promoter and origin; Neo/Kan ^R^ , neomycin- / kanamycin-resistance gene; scFv, single-chain variable fragment; V_H_, heavy chain variable region; V_L_, light chain variable region. (**B**) Flow cytometry analysis of the surface expression of scFv on HEK-293 T cells transfected with pDisplay-scFv vector. The red histogram depicts staining of transfected cells with anti-HA-tag-PE mAb, whereas the gray histogram represents transfected cells incubated with an irrelevant antibody as an isotype control. HA-tag, hemagglutinin-tag; scFv, single-chain variable fragment.

**Anti-KRAS^G12V^ CAR expression on JNL cells**

Count

Fluorescence


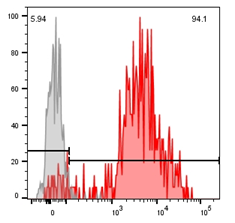

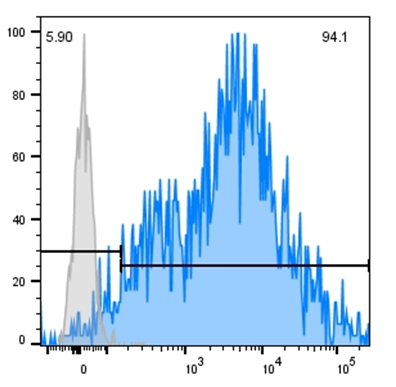


anti-hIgG

anti-Strep-tag II

Isotype control

**Supplementary Figure S5.** Flow cytometric analysis of the surface expression of anti-KRAS^G12V^ TCRm CARs on JNL cells. The red histogram illustrates the staining of the transduced CAR- IgFc JNL cells with an anti-human IgG-PE recognizing mAb (clone JDC-10). The blue histogram depicts CAR-STII JNL cells stained with an anti-Strep-tag II-Biotin mAb followed by SA-APC. The gray histograms represent CAR expressing cells stained with an irrelevant anti human IgG mAb to serve as an isotype control.

**Supplementary Table S1.** List of primers

| **Target fragment** | **Forward** | **Reverse** |
| --- | --- | --- |
| scFv D10-7 | 5′-ATAATGGCCCAGCCGGC CATG -3′ | 5′-ATCTGCCGCGGCGATGAGA CGGTGACCAGGG-3′ |
| Anti-CD3 scFv  (UCHT1) | 5′-CTCATCGGGCGGAGGTG  GGAGT-3′ | 5′-CTCGAGGCCTGCAGGAATT  CTTAATGGTGGTGGTGATGGT  GAGAG-3′ |
| scFv D10 | 5′-GCACTCTTGGCCGTATTG GCCCCGCCACCTGTGAC GGGAAACTTGTGGTAG-3′ | 5′-CGATCTTTGGCCTGTTTGGC CTTATACGCCAACAGCTCCAAC TACCACAAGTTTCC-3′ |
| hIgG-hCD28TMD-  hCD28STD-hCD3ζ | 5′-TAGTGGGGATCCCGCCG  AGC-3′ | 5′-GCGGCGCGCCGGCCCCCG  GATCTCTCGAGGATTAGC-3′ |
| Strep-tag II | 5′-GAGTAGTGCGGCCGCA  TGGAG-3′ | 5′- ACCCAAAATGCGGCCGCGC  T-3′ |
| hCD28TMD-hCD28STD-hCD3ζ | 5′-GGCCGCATTTTGGGTGC  TGGTGGT-3′ | 5′-GCGGCGCGCCGGCCCCCG  GATCTCTCGAGGATTAGC-3′ |

**Supplementary Table S2.** Tetramer and antibodies for flow cytometry

| **Antibodies / Tetramer** | **Clone / MHC type** | **Source** |
| --- | --- | --- |
| anti-human CD3-APC | UCHT1 | BD Biosciences |
| anti-human CD3-FITC | UCHT1 | BD Biosciences |
| anti-human CD56-PE | NCAM16.2 | BD Biosciences |
| anti-HA.11-tag-PE | 16B12 | BioLegend |
| anti-HLA-A2-PE | BB7.2 | BioLegend |
| anti-human IgG-PE | JDC-10 | SouthernBiotech |
| anti-mouse IgG2a-PE | G155-178 | SouthernBiotech |
| anti-Strep-tag II-Biotin | 5A9F9 | GenScript Biotech |
| Streptavidin-APC | NA | BioLegend |
| HLA-A*02:01-Tetramer-PE | NA | Tetramer Shop |
| anti-SIINFEKL/H2K^b^-APC | 25-D1.16 | eBioscience |
